# Effect and safety of drospirenone and ethinylestradiol tablets (II) for dysmenorrhea: A systematic review and meta-analysis

**DOI:** 10.3389/fmed.2022.938606

**Published:** 2022-12-15

**Authors:** Jinghua Shi, Jinhua Leng

**Affiliations:** ^1^Department of Obstetrics and Gynecology, Peking Union Medical College Hospital, Chinese Academy of Medical Sciences and Peking Union Medical College, Beijing, China; ^2^National Clinical Research Center for Obstetric & Gynecologic Diseases, Beijing, China

**Keywords:** ethinylestradiol/drospirenone, dysmenorrhea, systematic review, efficacy, safety

## Abstract

**Aim:**

This systematic review aimed to assess the efficacy and safety of Drospirenone and Ethinylestradiol Tablets (II) in the treatment of dysmenorrhea.

**Methods:**

Electronic databases, namely PubMed, Embase, Cochrane Controlled Register of Trials (CENTRAL), Scopus, Science, CBM, CNKI, Wanfang, and VIP, were searched before September 2022. Randomized controlled trials (RCTs), non-randomized controlled trials, cohort studies, case-control studies, and single-arm studies were included. Furthermore, the Cochrane Risk of Bias Tool for Systematic Reviews version 1 was used for the risk of bias assessment on RCTs. The Risk of Bias In Non-randomized Studies of Interventions (ROBINS-I) tool was used for risk of bias assessment on non-randomized studies. The risk ratio (RR) was calculated for dichotomous data. Mean difference (MD) or standardized MD (SMD) were used as the effect size for continuous data.

**Results:**

A total of 11 studies involving 2,251 participants with dysmenorrhea were included. When Drospirenone and Ethinylestradiol Tablets (II) conventional 24/4-day regimen was compared with placebo, the total efficiency rate (defined as pain symptom disappearing or being relieved) in Drospirenone and Ethinylestradiol Tablets (II) 24/4-day regimen group was higher than in placebo group (RR = 5.55, 95%CI: 2.48–12.39, *P* < 0.0001). No clear differences were found on risk of overall adverse events or specific adverse events. When Drospirenone and Ethinylestradiol Tablets (II) was compared with active control drugs, no clear differences were found on the total efficiency rate or visual analog scale (VAS) scores for dysmenorrhea and other related pain. The risk of overall adverse events decreased in Drospirenone and Ethinylestradiol Tablets (II) conventional 24/4-day regimen (13/53 vs. 66/148, RR = 0.55, 95%CI: 0.33–0.91) when compared with active control drugs group. When Drospirenone and Ethinylestradiol Tablets (II) flexible extended regimen was compared with conventional 24/4-day regimen, the number of days of dysmenorrhea (MD=−3.98, 95%CI: −5.69 to −2.27), and dysmenorrhea associated with unscheduled bleedings (MD = −1.6, 95%CI: −2.8 to −0.5), were fewer in flexible extended regimen. In addition, there were no differences found on risk of adverse events (including mood changes, spotting, headache, breast pain, nausea, and vomiting) between compared groups (*P* > 0.05).

**Conclusion:**

Drospirenone and Ethinylestradiol Tablets (II) could improve symptoms of dysmenorrhea and decrease other related pain symptoms. More high-quality evidence is needed to confirm the advantages.

**Systematic review registration:**

[https://www.crd.york.ac.uk/prospero/display_record.php?ID=CRD42021271605], identifier [CRD42021271605].

## Introduction

Dysmenorrhea refers to painful uterine cramps that occur during menstruation, concentrated in the lower abdomen, and may be accompanied by backache or other discomforts. It can be classified into primary dysmenorrhea (painful menstruation in the absence of pelvic pathology) and secondary dysmenorrhea (painful menses due to pelvic pathology or a recognized medical condition, such as endometriosis, adenomyosis, or pelvic inflammation) based on pathophysiology. There is still controversy over the pathogenesis of primary dysmenorrhea. However, current experiments and clinical studies found that excessive prostaglandin in the uterus is the main cause of primary dysmenorrhea. The estimated prevalence of dysmenorrhea is high (ranging from 45 to 93% in women of reproductive age, and highest in adolescent) (1). Dysmenorrhea has a huge negative impact on women’s quality of life, which is manifested as restrictions on daily activities, reduced academic performance in adolescents, and poor sleep quality. It can also negatively affect emotions, leading to anxiety and depression (1).

Combined oral contraceptives have been used in the treatment of dysmenorrhea since their introduction for general use in 1960 (2). The mechanism of action of COCs is to inhibit ovulation by estrogen and progesterone, thereby inhibiting the synthesis of prostaglandin (PG). The number of studies on the use of COCs for the treatment of dysmenorrhea has gradually increased with the further development with and growing experience of the application of COCs. Drospirenone and Ethinylestradiol Tablets (II) are a new generation of COC; each hormone-containing tablet contains 3 mg drospirenone and 20 μg ethinylestradiol. Firstly, it reduces estrogen production and lowers PG, vasopressin (VP), and oxytocin levels by inhibiting ovulation. Compared with the conventional 21/7 regimen, the 24/4 regimen results in greater inhibition of ovulation and lower fluctuations in hormone levels (3). At the same time, the 24/4 regimen results in lower estrogen dose (20 μg ethinylestradiol) and reduced exogenous estrogen stimulations. It can ensure good cycle control while decreasing estrogen-related side effects. Secondly, it can inhibit endometrial proliferation and reduce the production of PG in endometrium by inhibiting ovulation. Thirdly, it contains the unique progestogen drospirenone, which has a pharmacological profile similar to that of endogenous progesterone. It combines progestogenic, antimineralocorticoid, and antiandrogenic effects and does not exhibit any estrogenic, androgenic, or glucocorticoid effects, providing non-contraceptive benefits and decreasing estrogen-induced water and sodium retention as well as other adverse reactions (4, 5).

In recent years, most research data on Drospirenone and Ethinylestradiol Tablets (II) for relieving primary dysmenorrhea and secondary dysmenorrhea (such as endometriosis, adenomyosis, etc.) was from Japan, and there was no definite conclusion (6–13). There is still a lack of systematic and comprehensive evaluation of the effects (benefits and harms) of Drospirenone and Ethinylestradiol Tablets (II) for women with dysmenorrhea. The aim of this meta-analysis, therefore, was to assess the available evidence concerning the clinical effects and safety of Drospirenone and Ethinylestradiol Tablets (II) in dysmenorrhea treatment.

## Methods

The protocol for this review was registered in PROSPERO (registration number is CRD42021271605).

### Eligibility criteria

Studies that met the following criteria were included:

1) Study design: RCTs, non-randomized controlled trials, cohort studies, case-control studies, and single-arm studies, to show the full available evidence on Drospirenone and Ethinylestradiol Tablets (II);
2) Population: patients with primary dysmenorrhea and/or secondary dysmenorrhea (defined according to original studies) complicated with endometriosis or adenomyosis;
3) Intervention: Drospirenone (3 mg) and Ethinylestradiol (20 μg) Tablets (II);
4) Control: without Drospirenone and Ethinylestradiol Tablets (II);
5) Outcomes included total efficiency rate (=significant effective rate + effective rate), and VAS score of dysmenorrhea or other-related pain score (such as total pain score, total dysmenorrhea score, chronic pelvic pain, and dyspareunia), patient satisfaction using a measuring instrument which had been described in a peer-reviewed journal, HRQoL assessed by the 36-Item Short-Form Health Survey (SF-36) or other scales which had been described in a peer-reviewed journal, cycle control and changes in menstrual volume, and changes in size of endometriosis lesions. Safety was assessed by headache/migraine, nausea/vomiting, breast tenderness, mood changes, and spotting, etc.;
6) Published in English or Chinese.

### Literature search and study selection

An information specialist carried out the search in electronic databases [PubMed, Embase, Cochrane Controlled Register of Trials (CENTRAL), Scopus, Science CBM, CNKI, Wanfang, and VIP] in September 2022 using the keywords dysmenorrhea, menstrual pain, pelvic pain, ethinylestradiol/drospirenone, YAZ, and ethinylestradiol 20 μg plus drospirenone 3 mg (see Supplementary materials for details on the search strategy).

Two reviewers screened the search results independently according to eligibility criteria based on title and abstract. All potentially relevant citations were requested and inspected in full to identify final included studies. Any disagreement was resolved through discussion by two reviewers with assistance from a third party if necessary.

### Data extraction

Two reviewers independently extracted data from all the included studies using a standard data extraction form (Microsoft Excel). Any disagreement was resolved through discussion by two reviewers with assistance from a third party if necessary. Data extracted from RCTs and non-randomized controlled trials mainly included:

(1) Study information: author, year of publication, study design, number of sites, sample size (all and per group), number of groups, duration of follow-up, source of funding, etc.;
(2) Baseline characteristics of participants: classification of dysmenorrhea, age, BMI, menstrual characteristics, etc.;
(3) Description of interventions: treatment regimen and methods of administration (dose, frequency, and duration etc.);
(4) Outcome data: definitions of outcomes, drop-outs, timepoint of assessment, and results data (number of participants in each group with outcome events etc.).

For observational studies (cohort studies and case-control studies) or single-arm studies, the source of data and information of confounding control (non-adjusted results data, adjusted results data, and adjusted factors) was extracted in addition.

Primary outcomes in this review were defined as total efficiency rate and VAS score of dysmenorrhea or other-related pain score, and secondary outcomes were patient satisfaction, HRQoL, cycle control and changes in menstrual volume, changes in size of endometriosis lesions, and adverse events.

### Risk of bias assessment

The risk of bias assessment was independently performed by two reviewers. Any disagreement was resolved through discussion by two reviewers with assistance from a third party if necessary. The Cochrane Risk of Bias Tool for Systematic Reviews version 1 (14) was used for the risk of bias assessment on RCTs, including sequence generation, allocation concealment, blinding of participants and personnel, blinding of outcome assessment, incomplete outcome data, selective outcome reporting, and other bias. The Risk of Bias In Non-randomized Studies of Interventions (ROBINS-I) tool (15) was used for risk of bias assessment on non-randomized studies, including confounding, selection bias, bias in measurement classification of interventions, bias due to deviations from intended interventions, bias due to missing data, bias in measurement of outcomes, and bias in selection of reported result.

### Data analysis

The RR and its 95% CI were calculated for dichotomous data. MD or standardized MD (SMD) and its 95% CI were used as the effect size for continuous data. The significance level (α) was 0.05 for test for overall effect in meta-analysis. The Chi-square test and *I*^2^ were used to identify statistical heterogeneity. Substantial statistical heterogeneity is defined as *I^2^* ≥ 50% with a *P*-value of Chi-square test less than 0.1. When possible, different control groups (placebo vs. other active control drugs) were investigated in subgroup analysis. A publication bias test was not performed as the number of included studies was less than 10 (low power of this test) (16). RevMan 5.4 software was employed for all analyses. Meta-analyses were performed according to different study designs. Before meta-analyses, we fully discussed the similarity of included studies, and described the outcome data separately when meta-analyses could not be performed.

## Results

### Literature search results

**FIGURE 1:**
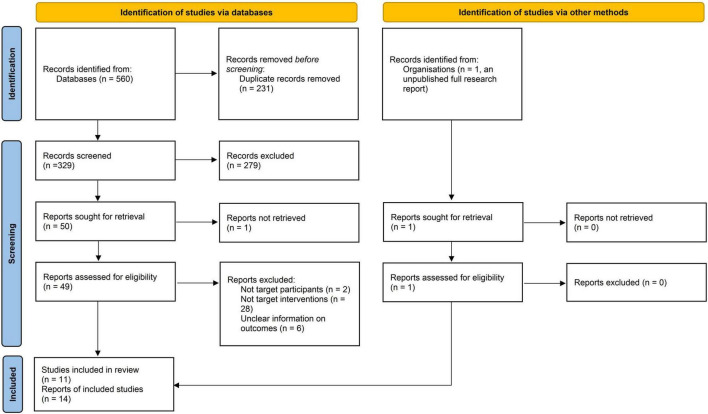
PRISMA flow diagram.

The database search yielded 352 records, and 295 records remained after duplicates were removed. After reading the titles and abstracts, 245 records which did not meet the inclusion criteria were excluded, and one additional record was excluded due to full-text unavailability. The remaining 50 potentially eligible records and one additional record (unpublished research report) were inspected in full, and subsequently 36 records were excluded. Finally, 11 studies with 14 references were included (6–13, 17–22). The PRISMA flow diagram is presented in Figure 1.

### Characteristics of included studies

Eleven studies (6–13, 17–22) were included involving 2,251 participants. There were 10 English studies (6–10, 12, 13, 17–22) and one Chinese study (11). Six studies (6–9, 11, 12, 17–19) were RCTs (*n* = 1534), one (13) was a non-randomized control study (*n* = 43), and four studies (10, 20–22) were single-arm studies (*n* = 674). The studies (6–13, 17–22) were conducted in Jordan (13), Japan (6–10, 12, 17, 20–22), China (11), Germany (18, 19), and the UK (18, 19). Two of the studies (11, 21) were single center studies, whereas nine studies (6–10, 12, 13, 17–20, 22) were multicenter studies. The study subjects were primary dysmenorrhea patients (three studies) (8, 11, 13, 18, 19), endometriosis patients (three studies) (12, 20, 22), and both primary dysmenorrhea and secondary dysmenorrhea patients (four studies) (6–10, 17). One study (20) did not specify the type of dysmenorrhea. The sample size for analyses ranged from 38 to 414. As reported, the average age ranged from 20.2 to 35.2 years, average BMI ranged from 20.3 to 22.4 kg/m^2^, and average length of menstrual cycle ranged from 27.7 to 29.5 days. Detailed information is shown on Table 1 and Supplementary Table 1.

**TABLE 1:** Characteristics of included studies.

Study ID	Study design	Participants	Intervention group		Control group		Outcomes
			**Description**	***n***	**Description**	***n***	
Al-Jefout and Nawaiseh (13)	Non-randomized controlled trial	PD patients	Drospirenone and Ethinylestradiol Tablets (II) conventional 24/4-day regimen	18	NET-A 5 mg/d continuous regimen	20	Dysmenorrhea score (VAS, 0–10), adverse events
Harada et al. (12)	RCT	Endometriosis patients	Drospirenone and Ethinylestradiol Tablets (II) flexible extended regimen	130	Placebo Dienogest 2 mg/d	128 53	Endometriosis-associated pelvic pain (VAS, 0–100), dyspareunia, size and number of endometriomas, induration in the cul-de-sac, limitation of uterine mobility, pelvic tenderness, patient satisfaction, adverse events
Liu and Lin (11)	RCT	PD patients	Drospirenone and Ethinylestradiol Tablets (II) conventional 24/4-day regimen	53	Placebo	30	Total efficiency rate. dysmenorrhea score (VAS, 0–10), adverse events
					Desogestrel/ethinylestradiol tablets 150 μg/30 μg	51	
					Cyproterone/ethinylestradiol tablets 2 mg/35 μg	48	
					Drospirenone/ethinylestradiol tablets 3 mg/30 μg	49	
Momoeda et al. (10)	Single-arm	Dysmenorrhea patients (PD + SD)	Drospirenone and Ethinylestradiol Tablets (II) conventional 24/4-day regimen	186	NA	NA	Health-related quality of life score
NCT00461305 (8, 9)	RCT	Dysmenorrhea patients (PD + SD)	Drospirenone and Ethinylestradiol Tablets (II) conventional 24/4-day regimen	349	Drospirenone/ethinylestradiol tablets 3 mg/30 μg conventional 24/4-day regimen	65	Dysmenorrhea score (VAS, 0–100), pelvic pain score (VAS, 0–100), adverse events
NCT00511797 (7)	RCT	Dysmenorrhea patients (PD + SD)	Drospirenone and Ethinylestradiol Tablets (II) conventional 24/4-day regimen	61	Placebo	58	Dysmenorrhea score (VAS, 0–100), adverse events
NCT01892904 (6, 17)	RCT	Dysmenorrhea patients (PD + SD)	Drospirenone and Ethinylestradiol Tablets (II) flexible extended regimen	105	Drospirenone and Ethinylestradiol Tablets (II) conventional 24/4-day regimen	107	Number of days of dysmenorrhea, number of days of pelvic pain, patient satisfaction, adverse events
Strowitzki et al. (18, 19)	RCT	Moderate to severe PD patients	Drospirenone and Ethinylestradiol Tablets (II) flexible extended regimen	115	Drospirenone and Ethinylestradiol Tablets (II) conventional 24/4-day regimen	108	Number of days of dysmenorrhea, number of days of pelvic pain, patient satisfaction, adverse events
Takeda et al. (20)	Single-arm	Dysmenorrhea patients with PMS/PMDD symptoms (unknown)	Drospirenone and Ethinylestradiol Tablets (II) conventional 24/4-day regimen	39	NA	NA	Degree of dysmenorrhea
Tanaka et al. (21)	Single-arm	Endometriosis patients	Drospirenone and Ethinylestradiol Tablets (II) conventional 24/4-day regimen	46	NA	NA	Dysmenorrhea score (VAS, 0–100), chronic pelvic pain, dyspareunia, size of ovarian endometrioma, tenderness and induration in the cul-de-sac, adverse events
Taniguchi et al. (22)	Single-arm	Endometriosis patients	Drospirenone and Ethinylestradiol Tablets (II) conventional 24/4-day regimen	49	NA	NA	Size of ovarian endometrioma (the maximum diameter and volume of the ovarian endometrioma), dysmenorrhea score (VAS, 0–100), serious adverse events

d, day; NA, not applicable; NET-A, norethisterone acetate; NR, not reported; PD, primary dysmenorrhea; PMS, premenstrual syndrome; PMDD, premenstrual dysphoric disorder; SD, secondary dysmenorrhea; VAS, visual analog scale.

Of the included eleven studies (6–13, 17–22), five studies (7–9, 11–13) compared Drospirenone and Ethinylestradiol Tablets (II) with placebo or active control drugs, two studies (6, 17–19) compared Drospirenone and Ethinylestradiol Tablets (II) flexible extended regimen and conventional 24/4-day regimens, and four studies (10, 20–22) were single-arm studies of Drospirenone and Ethinylestradiol Tablets (II). Table 1 shows the reported outcomes of the various studies which were pre-defined in this review.

### Risk of bias in included studies

Among the six included RCTs (6–9, 11, 12, 17–19), the overall risk of bias was moderate. Of the studies, 50% (8, 9, 11, 12) used appropriate randomization methods and were rated as low risk. Two studies (6, 17–19) were open-label studies, and rated as high risk for selection bias, performance, and assessment bias. Only one study had no missing outcome data (11), whereas drop-outs were present in the other five studies (6–9, 12, 17–19), which were rated as unclear risk of attrition bias. No obvious bias existed on other domains.

The ROBINS-I tool (15) was used for risk of bias assessment of five included non-randomized studies (10, 13, 20–22). Among the five studies (10, 13, 20–22), one study (13) adjusted for important confounding factors and the confounding bias was rated as low, whereas four studies (10, 20–22) were single-arm studies in which the confounding bias was rated as moderate. Two studies (10, 20) had high drop-out rates (>20%) and bias due to missing data was rated as high. Two studies (13, 21) had low drop-out rates and bias due to missing data was rated as moderate. There were no missing data in one study (22), so the bias due to missing data were rated as low. The selection bias, bias in measurement classification of interventions, bias due to deviation from intended interventions, bias due to missing data, bias in measurement of outcomes, and bias in selection of reported result of all studies (10, 13, 20–22) were low. Table 2 shows the details of the specific results of assessment of RCTs and non-randomized studies.

**TABLE 2:** Bias risk assessment results.

**RCT**								
**Study ID**	**Sequence generation**	**Allocation concealment**	**Blinding of participants**	**Blinding of personnel**	**Blinding of outcome assessment**	**Incomplete outcome data**	**Selective outcome reporting**	**Other bias (baseline status)**
Harada et al. (12)	Low risk	Low risk	Unclear	Unclear	Unclear	Unclear	Low risk	Low risk
Liu and Lin (11)	Low risk	Unclear	Unclear	Unclear	Unclear	Low risk	Low risk	Low risk
NCT00461305 (8, 9)	Low risk	Unclear	Low risk	High risk	Unclear	Unclear	Low risk	Low risk
NCT00511797 (7)	Unclear	Unclear	Low risk	Low risk	Unclear	Unclear	Low risk	Low risk
NCT01892904 (6, 17)	Unclear	High risk	High risk	High risk	High risk	Unclear	Low risk	Low risk
Strowitzki et al. (18, 19)	Unclear	High risk	High risk	High risk	High risk	Unclear	Low risk	Low risk
**Non-randomized studies**								
**Study ID**	**Confounding**	**Selection bias**	**Bias in measurement classification of interventions**	**Bias due to deviations from intended interventions**	**Bias due to missing data**	**Bias in measurement of outcomes**	**Bias in selection of reported result**	**Overall**
Al-Jefout and Nawaiseh (13)	Low risk	Low risk	Low risk	Low risk	Moderate risk	Low risk	Low risk	Moderate risk
Momoeda et al. (10)	Moderate risk	Low risk	Low risk	Low risk	High risk	Low risk	Low risk	High risk
Takeda et al. (20)	Moderate risk	Low risk	Low risk	Low risk	High risk	Low risk	Low risk	High risk
Tanaka et al. (21)	Moderate risk	Low risk	Low risk	Low risk	Moderate risk	Low risk	Low risk	Moderate risk
Taniguchi et al. (22)	Moderate risk	Low risk	Low risk	Low risk	Low risk	Low risk	Low risk	Low risk

### Effects of interventions

As some studies included both primary dysmenorrhea and secondary dysmenorrhea patients (6–10, 17) or the classification of dysmenorrhea was unknown (20), the study population could not be divided into subgroup datasets. Hence, analyses were performed according to different comparisons in included studies.

### Comparison of drospirenone and ethinylestradiol tablets (II) versus placebo or active control drugs

#### Total efficiency rate

Only one RCT [Liu and Lin (11)] was identified on this outcome. In this RCT, total efficiency rates for patients who received the Drospirenone and Ethinylestradiol Tablets (II) conventional 24/4-day regimen, placebo, or active control drugs were assessed. Significantly effective was defined as the patient’s symptoms significantly improving without dysmenorrhea; effective as the patient’s symptoms and pain significantly reducing, without relying on analgesics; no clinical response if the patient’s symptoms did not improve or even worsened. Total efficiency rate = significant effective rate + effective rate. Results showed that the total efficiency rate after 6-cycle treatment of the Drospirenone and Ethinylestradiol Tablets (II) conventional 24/4-day regimen was higher than that in placebo group (RR = 5.55, 95%CI: 2.48–12.39, *P* < 0.0001), but there was no clear difference on this outcome for Drospirenone and Ethinylestradiol Tablets (II) conventional 24/4-day regimen versus active control drugs group (RR = 0.99, 95%CI: 0.91–1.08, *P* = 0.85) (Supplementary Table 2).

#### Dysmenorrhea

Three studies (7, 11, 13) reported VAS score for dysmenorrhea before and after treatment. Two were RCTs (7, 11), whereas one (13) was a non-randomized controlled trial.

The non-randomized controlled trial, Al-Jefout and Nawaiseh (13), used a 0–10 point VAS to evaluate dysmenorrhea improvement before and after 3- and 6-month treatment with Drospirenone and Ethinylestradiol Tablets (II) conventional 24/4-day regimen or norethisterone acetate 5 mg/d continuous regimen in primary dysmenorrhea patients. Results showed that there was no clear difference in VAS score reduction between the compared groups (3-month: MD = −0.54, 95%CI: −1.18 to 0.10, *P* = 0.10; 6-month: MD = −0.21, 95%CI: −0.91 to 0.49, *P* = 0.56).

The remaining two RCTs (7, 11) reported the mean and SD of VAS scores before and after treatment in the Drospirenone and Ethinylestradiol Tablets (II) conventional 24/4-day regimen and control groups. Results showed that the Drospirenone and Ethinylestradiol Tablets (II) conventional 24/4-day regimen was superior to the placebo in reducing VAS score for dysmenorrhea (SMD=−1.05, 95%CI: −1.39 to −0.71; *P*<0.00001). There was no clear difference in reduction of VAS score for dysmenorrhea when Drospirenone and Ethinylestradiol Tablets (II) conventional 24/4-day regimen compared with active control drugs (SMD = −0.01, 95%CI: −0.42 to 0.40; *P* = 0.96). The results for reduction in VAS score for dysmenorrhea showed that differences between subgroups were statistically significant (*P* = 0.0001), suggesting that different controls may be the main source of heterogeneity (Supplementary Figure 1).

#### Pelvic pain and other pain-related outcomes

Two RCTs (8, 9, 12) reported VAS score for pelvic pain and other pain score. Harada et al. (12) found that at week 24, Drospirenone and Ethinylestradiol Tablets (II) flexible extended regimen significantly reduced VAS score for endometriosis-associated pelvic pain compared to placebo (least squares mean difference=−26.3, 95%CI: −31.6 to 20.9; *P*<0.0001). In addition, descriptive results showed that Drospirenone and Ethinylestradiol Tablets (II) flexible extended regimen has superior effects than placebo in reduction in pain measures associated with endometriosis (including pelvic pain during menstrual/non-menstrual or withdrawal/non-withdrawal bleeding period, severest dyspareunia, severest defecation pain, severest pelvic pain except for dyspareunia and defecation pain, severest pelvic pain that is always present or lasts for a long time, and severest pelvic pain that lasts only a short time and then disappears). In NCT00461305 2007 (8, 9), there were no clear differences in VAS score for pelvic pain during non-menstrual period between the Drospirenone and Ethinylestradiol Tablets (II) conventional 24/4-day regimen and drospirenone/ethinylestradiol tablets 3 mg/30 μg 24/4-day regimen after 6-cycle treatment (at day 168), (MD=−4.90, 95%CI: −11.36 to 1.56, *P* = 0.14). In addition, descriptive results showed that the mean (SD) change in VAS score for pelvic pain during non-menstrual period compared with baseline was −10.9 (24.55) points in Drospirenone and Ethinylestradiol Tablets (II) conventional 24/4-day regimen group after 13-cycle treatment.

With regard to other pain scores, Harada et al. (12) found that Drospirenone and Ethinylestradiol Tablets (II) flexible extended regimen is superior to placebo in reducing dyspareunia, defecation pain, average pain-related VAS scores, and the number of days with pain at week 24. With reference to improvement in pelvic tenderness, the proportion of patients with “none” and “mild” pain increased from 57.7 to 85.6% [Drospirenone and Ethinylestradiol Tablets (II) flexible extended regimen group] and from 63.3 to 64.9% (placebo group). The other RCT (NCT00461305 2007) (8, 9) employed the 0–100 point VAS to evaluate other pain at times other than during menstruation in patients who received Drospirenone and Ethinylestradiol Tablets (II) conventional 24/4-day regimen or drospirenone/ethinylestradiol tablets 3 mg/30 μg conventional 24/4-day regimen. Results showed that after 6-cycle treatment (at day 168), there were no clear differences in reduction of pain (VAS scores) at times other than during menstruation between the compared groups (MD = −5.30, 95%CI: −12.37 to 1.77, *P* = 0.14). In addition, after 13-cycle treatment, the mean (standard deviation, SD) change in VAS score for other pain (VAS scores) at times other than during menstruation compared with baseline was −41.6 (24.70) points in the Drospirenone and Ethinylestradiol Tablets (II) conventional 24/4-day regimen group.

#### Changes in endometriosis lesions

Descriptive results reported in one RCT [Harada et al. (12)] showed the number of endometriomas at week 24 slightly reduced in Drospirenone and Ethinylestradiol Tablets (II) flexible extended regimen group compared with baseline (2.0 ± 1.5 vs. 1.2 ± 1.0), but similar results were not observed in placebo group (1.3 ± 0.9 vs. 1.4 ± 0.8). The geometric mean size of endometriomas decreased in Drospirenone and Ethinylestradiol Tablets (II) flexible extended regimen group compared with baseline (29.87 ± 1.58 mm vs. 24.33 ± 1.79 mm), but similar results were not observed in placebo group (28.86 ± 1.57 mm vs. 28.84 ± 1.59 mm). There was an increase from baseline in the proportion of patients with “none” or “mild” induration in the cul-de-sac (63.1–85.6% vs. 71.9–74.8%), “none” or “mild” limitation of uterine mobility (70.8–81.7% vs. 71.1–72.1%) in Drospirenone and Ethinylestradiol Tablets (II) flexible extended regimen and placebo groups, respectively.

#### Patient satisfaction

In one RCT [Harada et al. (12)], patients were required to score overall treatment satisfaction by choosing one of seven categories form very much satisfied to very much dissatisfied at week 24. Descriptive results showed that 43.1% in Drospirenone and Ethinylestradiol Tablets (II) flexible extended regimen group and 10.3% in placebo group were “very much satisfied/much satisfied.”. Overall, 38.1% of patients in placebo group were “neither satisfied nor dissatisfied” with the overall treatment.

#### Safety

Five included studies (7–9, 11–13) reported adverse events during treatment and follow-up periods. Results from Al-Jefout and Nawaiseh (13) showed that there was no significant difference (*P* = 0.745) in the weight gain (kg, mean ± SD) in Drospirenone and Ethinylestradiol Tablets (II) conventional 24/4-day regimen group (0.22 ± 1.06) and norethisterone acetate 5 mg/d continuous regimen (0.35 ± 1.3). Two patients (both cases occurred 3 months after starting treatment) were found with deep vein thrombosis in Harada et al. (12), and no clear differences were found when compared with placebo (2/130 vs. 0/128, RR = 4.92, 95%CI: 0.24–101.56, *P* = 0.30).

Only one RCT [Liu and Lin (11)] reported the number of overall adverse events for each group. This trial found no clear differences between Drospirenone and Ethinylestradiol Tablets (II) conventional 24/4-day regimen and placebo group (13/53 vs. 2/30, RR = 3.68, 95%CI: 0.89–15.22), and the risk of overall adverse events decreased in Drospirenone and Ethinylestradiol Tablets (II) conventional 24/4-day regimen group (13/53 vs. 66/148, RR = 0.55, 95%CI: 0.33–0.91) when compared with active control drugs. No clear differences were found on risk of overall adverse events or specific adverse events (including nausea, vomiting, distending pain in breast, spotting, and headache) between Drospirenone and Ethinylestradiol Tablets (II) and placebo. Only one RCT [Liu and Lin (11)] found that the risk of overall adverse events decreased in Drospirenone and Ethinylestradiol Tablets (II) conventional 24/4-day regimen group (13/53 vs. 66/148, RR = 0.55, 95%CI: 0.33–0.91) when compared with active control drugs. No clear differences were found on risk of other specific adverse events (including mood changes, spotting, headache, breast pain, nausea, and vomiting) between compared groups. (Supplementary Table 3 shows the details of these adverse events).

### Comparison of drospirenone and ethinylestradiol tablets (II) flexible extended regimen versus conventional 24/4-day regimen

Two RCTs (6, 17, 19) compared Drospirenone and Ethinylestradiol Tablets (II) flexible extended regimen with conventional 24/4-day regimen. Both RCTs (6, 17, 19) reported the number of days of dysmenorrhea, number of days of pelvic pain, and patient satisfaction.

#### Number of days of dysmenorrhea

The results of meta-analysis (6, 17, 19) showed that the number of days of dysmenorrhea were fewer in Drospirenone and Ethinylestradiol Tablets (II) flexible extended regimen group than that in conventional 24/4-day regimen group (MD = −3.98, 95%CI: −5.69 to −2.27, *P* < 0.00001) (Supplementary Figure 2).

NCT01892904 2017 (6, 17) found that the number of days with dysmenorrhea (defined as any spasmodic pelvic pain or lower abdominal pain with possible radiation toward back or thighs recorded during withdrawal and/or a menstrual bleeding episode) associated with unscheduled bleeding was fewer in Drospirenone and Ethinylestradiol Tablets (II) flexible extended regimen group than that in conventional 24/4-day regimen group (MD = −1.6, 95%CI: −2.8 to −0.5) at day 140 after treatment. In addition, there were no clear differences in the number of days with dysmenorrhea (at least “moderate”) (MD = −1.2, 95%CI: −2.6 to 0.1) and days with dysmenorrhea associated with withdrawal bleedings (MD = −1.8, 95%CI: −4.4 to 0.9).

#### Number of days of pelvic pain

NCT01892904 2017 (6, 17) showed the average number of days with pelvic pain independent of vaginal bleeding at day 140 after treatment was fewer in Drospirenone and Ethinylestradiol Tablets (II) flexible extended regimen group than that in conventional 24/4-day regimen group, but the difference was not statistically significant (MD = −3.5, 95%CI: −8.3 to 1.4).

Strowitzki et al. (18, 19) showed the number of days with pelvic pain independent of vaginal bleeding at day 140 after treatment was fewer in Drospirenone and Ethinylestradiol Tablets (II) flexible extended regimen group than that in conventional 24/4-day regimen group (MD = −3.4, 95%CI: −5.9 to −0.9).

#### Patient satisfaction

Patients in NCT01892904 2017 (6, 17) were required to score their satisfaction (scale from 1 = very much satisfied to 7 = very much dissatisfied, where 4 = neither satisfied nor dissatisfied), and the results showed that the proportion of patients who were “very much satisfied” and “much satisfied” were similar between Drospirenone and Ethinylestradiol Tablets (II) flexible extended regimen group and conventional 24/4-day regimen group (54.3% vs. 50.9%). In the Strowitzki study (18, 19), patients were required to score satisfaction (seven-point scale ranging from 1 = very much satisfied, to 7 = very much dissatisfied), and the proportion of patients in Drospirenone and Ethinylestradiol Tablets (II) flexible extended regimen and conventional 24/4-day regimen groups who were “very much satisfied” (21.7% vs. 24.1%) or “much satisfied” (54.8% vs. 50.0%) and the proportion of patients with “minimally satisfied/neither satisfied nor dissatisfied” was close (17.4% vs. 14.8%). Whereas the proportion of patients who were “minimally dissatisfied/much or very much dissatisfied” was 6.1% in flexible extended regimen group and 11.0% in conventional 24/4-day regimen group.

#### Safety

Two RCTs (6, 17–19) reported adverse events during treatment and follow-up period of Drospirenone and Ethinylestradiol Tablets (II) flexible extended regimen and conventional 24/4-day regimens (Supplementary Table 4 and Supplementary Figure 3). Although Strowitzki et al. (18, 19) showed fewer participants with flexible extended regimen experienced headache than those with conventional 24/4-day regimen, the results of meta-analysis (29/220 vs. 42/215, RR = 0.57, 95%CI: 0.15–2.14) indicated no clear difference on risk of headache. No clear differences were found on risk of other specific adverse events (including breast pain and vomiting) between compared groups.

### Results of single-arm studies on dysmenorrhea treatment with drospirenone and ethinylestradiol tablets (II)

Four single-arm studies (10, 20–22) investigated Drospirenone and Ethinylestradiol Tablets (II) conventional 24/4-day regimen on dysmenorrhea treatment.

Momeda et al. (10) employed the Japanese language version of the 36-Item Short-Form Health Survey version 2.0 (SF-36v2, high score defines a more favorable health state) that consists of eight domains (physical functioning, role physical, bodily pain, general health, vitality, social functioning, role emotional, and mental health) to evaluate improvement in HRQoL before and after 6-cycle and 8-cycle treatment with the Drospirenone and Ethinylestradiol Tablets (II) conventional 24/4-day regimen. Results showed that significant increases (mean change ± SD) in the scores from baseline of eight domains were identified (*P* < 0.001): physical functioning (1.4 ± 5.7), role physical (3.2 ± 8.1), bodily pain (7.8 ± 10.0), general health (3.0 ± 7.0), vitality (2.7 ± 8.1), social functioning (3.5 ± 9.8), role emotional (3.3 ± 9.2), and mental health (3.0 ± 7.3). Moreover, after 6- to 8-cycle treatment, scores (mean ± SD) for general health domain (52.0 ± 9.0) and physical component summary (51.6 ± 9.1) of participants were significantly higher than those in the Japanese general population (*P* = 0.008 and *P* = 0.033, respectively).

Takeda et al. (20) reported VAS score for dysmenorrhea before and after 6-cycle treatment with the Drospirenone and Ethinylestradiol Tablets (II) conventional 24/4-day regimen in dysmenorrhea patients with PMS/PMDD symptoms. Results showed that Drospirenone and Ethinylestradiol Tablets (II) conventional 24/4-day regimen could significantly reduce the degree of dysmenorrhea in patients (baseline versus after treatment: 6.57 ± 1.96 versus 2.65 ± 1.78, *P* < 0.001).

Tanaka et al. (21) reported VAS score on assessment as follows: (1) improvement in dysmenorrhea, pelvic pain, dyspareunia, and tenderness in the cul-de-sac; (2) changes in the size of the ovarian endometrioma; and (3) the proportion of patients with induration in the cul-de-sac before and after 3- and 6-cycle treatment with Drospirenone and Ethinylestradiol Tablets (II) conventional 24/4-day regimen. Results showed that after 3-cycle treatment, VAS score for dysmenorrhea (median [interquartile range, IQR]) decreased from 71 (50–80) to 30 (12.5–55); VAS score for chronic pelvic pain decreased from 30 (10–60) to 10 (0–30); and VAS score for dyspareunia decreased from 10 (0–35) to 0 (0–20). Tenderness in the cul-de-sac significantly decreased compared with pre-treatment assessment (*P* = 0.001). The proportion of patients with induration in the cul-de-sac decreased from a baseline value of 49% (22/45) to 27% (10/37). All differences were statistically significant (*P* < 0.05). After 6-cycle treatment, VAS score for dysmenorrhea decreased to 24 (10–40); VAS score for chronic pelvic pain decreased to 5 (0–21); and VAS score for dyspareunia decreased to 0 (0–10). Tenderness in the cul-de-sac significantly decreased compared with pre-treatment assessment (*P* < 0.001). The proportion of patients with induration in the cul-de-sac decreased to 18% (7/39). All differences were statistically significant (*P* < 0.05) as well. As reported, 35 patients with ovarian endometrioma in this study had their endometrioma diameter [median (IQR)] decrease from 34.5 mm (0–44 mm) at baseline to 25.5 mm (0–36 mm) (*P* = 0.015) after 3-cycle treatment with Drospirenone and Ethinylestradiol Tablets (II) conventional 24/4-day regimen and decrease to 9.5 mm (0–31 mm) after 6-cycle treatment (*P* = 0.003).

Taniguchi et al. (22) reported VAS score for endometriosis-associated dysmenorrhea improvement assessment before and after 1-, 3-, and 6-cycle treatment with the Drospirenone and Ethinylestradiol Tablets (II) conventional 24/4-day regimen. Vaginal ultrasound was used to evaluate changes in size of ovarian endometrioma before and after treatment. Results showed that the median dysmenorrhea VAS score decreased from baseline (68 mm) to 27, 10, and 10 mm after 1-, 3-, and 6-cycle treatment, respectively. All differences were statistically significant (*P* < 0.001). The maximum diameter of ovarian endometrioma significantly decreased after 3-cycle (median: 31.0 mm versus pre-treatment 35.0 mm) and 6-cycle (median:29.0 mm versus pre-treatment) treatment. Similarly, the volume of ovarian endometrioma was also reduced after 3-cycle (median: 10.6 cm^3^ vs. pre-treatment: 16.5 cm^3^) and 6-cycle (6.7 cm^3^ vs. pre-treatment) treatment. All differences were statistically significant (*P* < 0.001). In addition, no new ovarian endometriomas were identified at 6-cycle treatment.

Two studies reported adverse events (20, 21). Results in Takeda et al. (20) showed that weight gain and water-retention symptoms were significantly improved only after the third cycle, but not after the sixth cycle when comparing with baseline. Intense press coverage about a death caused by thrombosis associated with Drospirenone and Ethinylestradiol Tablets (II) conventional 24/4-day regimen was reported during the course of Takeda et al. (20). One patient in Tanaka et al. (21) was lost to follow-up due to weight gain. No serious adverse drug reaction occurred (Supplementary Table 5).

## Discussion

For dysmenorrhea and dysmenorrhea-related disorders, COC was recommended as the first-line treatment for the relief of primary or secondary dysmenorrhea in global relevant consensus and guidelines. COCs are effective in relieving dysmenorrhea and reducing menstrual volume by reducing the secretion of PG and VP through inhibition of ovulation and anti-proliferation of the endometrium (23). Drospirenone and Ethinylestradiol Tablets (II), a new generation of COC, has both anti-mineralocorticoid and anti-androgen activities with low doses of ethinyl estradiol. It has been marketed in Japan for the indication of “dysmenorrhea” for more than 10 years. A real-world study in Japan showed a significant improvement in physiological, social, and psychological HRQoL in dysmenorrhea patients after a cyclic regimen of Drospirenone and Ethinylestradiol Tablets (II) (10). In a single-arm, open-label, interventional, multicenter, post-authorization study of Drospirenone and Ethinylestradiol Tablets (II) in China (24), the dysmenorrhea subgroup included a total of 526 subjects with dysmenorrhea in the 6 months prior to enrollment, and the severity of menstrual pain was assessed using the VAS score (0–100) at all four visits during 6 cycles of Drospirenone and Ethinylestradiol Tablets (II) 24/4-day regimen. Results showed a continuous decrease in menstrual pain at each visit when compared to baseline. The mean change in pain severity from baseline was −16.6 (*SD* = 22.9) at visit 2 (cycle 1), −28.1 (*SD* = 25.4) at visit 3 (cycle 4), and −31.2 (*SD* = 26.5) at visit 4 (cycle 6) (25). The results showed the effectiveness of Drospirenone and Ethinylestradiol Tablets (II) in Chinese women with dysmenorrhea. Another study in Japan involved 315 patients (mean age 28.9 years) with dysmenorrhea and 262 patients (mean age 31.3 years) with endometriosis showed that ethinylestradiol and drospirenone could improve QOL outcomes for patients with dysmenorrhea or endometriosis-associated pelvic pain (26). Evidence in our systematic review indicated that Drospirenone and Ethinylestradiol Tablets (II) could relieve dysmenorrhea and reduce the diameter of ovarian cysts when compared with before treatment, which was consistent with the above results. When comparing with placebo, Drospirenone and Ethinylestradiol Tablets (II) also has advantages on dysmenorrhea relieving, while the superior efficiency is still uncertain when comparing with other active controls.

In terms of safety, Drospirenone Ethinylestradiol Tables (II) shortened the hormone-free interval, providing good tolerability with −20μg of ethinylestradiol and 3 mg of drospirenone. An international multicenter study of Drospirenone Ethinylestradiol Tables (II) showed that only 0.7% of women discontinued study medication due to irregular bleeding, which was lower than other COCs (2–13%), in particular lower than other COCs containing 20 μg ethinylestradiol (13% discontinuation rate of norethindrone acetate/ethinylestradiol 1 mg/20 μg; 6% discontinuation rate of desogestrel/ethinylestradiol 150 μg/20 μg) (27). Adverse events reported in this study were predominantly mild to moderate. Reported treatment-related adverse events, such as headache (6.5%), breast pain (6.3%), and nausea (2.5%), were consistent with the known adverse events reported on other COCs (27). In the single-arm, open-label, interventional, multicenter, post-authorization study of Drospirenone and Ethinylestradiol Tablets (II) in China, good safety and no serious adverse events occurred. Incidence of common adverse events was 12.9%. The most common treatment-related adverse events were nausea, breast tenderness, and headache, most of which were mild and subsequently relieved, similar to international data (25). Our systematic review also showed that Drospirenone Ethinylestradiol Tables (II) had a good safety profile (such as low risk in thrombus, weight gain, or water-retention), good tolerability, and a low overall risk of adverse events compared to other positive controls. The study in Japan (26) also validated the safety and efficacy of Drospirenone Ethinylestradiol in patients with Endometriosis-associated pelvic pain and Dysmenorrhea. The reasons may be that the low estrogenic dose of Drospirenone Ethinylestradiol Tables (II) could reduce estrogen-related adverse effects; the pharmacological activity of Drospirenone Ethinylestradiol Tables (II) itself (no androgenic activity, anti-androgenic activity, and anti-mineralocorticoid activity), and the more stable overall sex hormone levels resulting from the 24/4-day regimen may also reduce the overall incidence of adverse events. Overall, the adverse events of Drospirenone Ethinylestradiol Tables (II) were tolerable and could be used in clinical practice.

This systematic review employed a scientific and rigorous evidence-based approach. We developed comprehensive search strategies and performed the search of English and Chinese databases. There were some limitations of this review. Only English and Chinese studies were included. A small number of included studies and small sample size on each outcome affected the reliability of the study results to some extent, and potential publication bias could not be assessed. In addition, there are omissions in some of the outcome information and various assessment methods used in included studies, which limited data analysis. This somewhat limited the applicability of the study results.

In conclusion, the results of this systematic review further confirmed the effectiveness of Drospirenone Ethinylestradiol (II) Tablets in consistently improving dysmenorrhea, pelvic pain, and other pain-related outcomes, as well as improving endometriosis lesions. In future, more rigorous and standardized RCTs or observational studies are needed. High-quality analysis and reporting should also be conducted to further validate and improve the results of the review.

## Data availability statement

The original contributions presented in this study are included in the article/Supplementary material, further inquiries can be directed to the corresponding author.

## Author contributions

Both authors listed have made a substantial, direct, and intellectual contribution to the work, and approved it for publication.

## References

[1] Bernardi M Lazzeri L Perelli F Reis F Petraglia F. Dysmenorrhea and related disorders. *F1000Res.* (2017) 6:1645. 10.12688/f1000research.11682.1 28944048 PMC5585876

[2] Proctor M Roberts H Farquhar C. Combined oral contraceptive pill (OCP) as treatment for primary dysmenorrhoea. *Cochrane Database Syst Rev.* (2001) 4:Cd002120. 10.1002/14651858.CD002120 11687142

[3] Tian Y Zhao Z. Clinical application and evaluation of drospirenone and ethinylestradiol tablets (II). *Drug Eval.* (2017) 14:42–6.

[4] Krattenmacher R. Drospirenone: pharmacology and pharmacokinetics of a unique progestogen. *Contraception.* (2000) 62:29–38. 10.1016/S0010-7824(00)00133-5 11024226

[5] Rübig A. Drospirenone: a new cardiovascular-active progestin with antialdosterone and antiandrogenic properties. *Climacteric.* (2003) 6(Suppl. 3):49–54. 15018248

[6] Momoeda M Kondo M Elliesen J Yasuda M Yamamoto S Harada T. Efficacy and safety of a flexible extended regimen of ethinylestradiol/drospirenone for the treatment of dysmenorrhea: a multicenter, randomized, open-label, active-controlled study. *Int J Womens Health.* (2017) 9:295–305. 10.2147/IJWH.S134576 28496369 PMC5422539

[7] NCT00511797,. *SH T00186 Phase II/III Optimal Drospirenone (DRSP) Dose Finding and Placebo-controlled Comparative Study.* (2007). Available online at: https://clinicaltrials.gov/ct2/show/NCT00511797 (accessed January 26, 2017).

[8] NCT00461305. *Clinical Study Report No.A41541 Amendment 2_April 5*. ClinicalTrials.gov (2010).

[9] NCT00461305. *Safety Study of Ethinylestradiol/Drospirenone in Dysmenorrhea.* (2007). Available online at: https://clinicaltrials.gov/ct2/show/NCT00461305 (accessed January 24, 2013).

[10] Momoeda M Akiyama S Tanaka K Suzukamo Y. Quality of life in Japanese patients with dysmenorrhea treated with ethinylestradiol 20 μg/drospirenone 3 mg in a real-world setting: an observational study. *Int J Womens Health.* (2020) 12:327–38. 10.2147/IJWH.S238460 32440228 PMC7210450

[11] Liu L Lin Z. Effect of different combinated of oral contraceptives in the treatment of dysmenorrhea. *Chin J Drug Eval.* (2019) 36:300–4.

[12] Harada T Kosaka S Elliesen J Yasuda M Ito M Momoeda M. Ethinylestradiol 20 mug/drospirenone 3 mg in a flexible extended regimen for the management of endometriosis-associated pelvic pain: a randomized controlled trial. *Fertil Steril.* (2017) 108:798–805. 10.1016/j.fertnstert.2017.07.1165 28911925

[13] Al-Jefout M Nawaiseh N. Continuous norethisterone acetate versus cyclical drospirenone 3 mg/ethinyl estradiol 20 mug for the management of primary dysmenorrhea in young adult women. *J Pediatr Adolesc Gynecol.* (2016) 29:143–7. 10.1016/j.jpag.2015.08.009 26342733

[14] Higgins JA Sterne J. Assessing risk of bias in included studies. In: Higgins J Green S editors. *Cochrane Handbook for Systematic Reviews of Interventions Version 5.1.0.* London: The Cochrane Collaboration (2011).

[15] Sterne J Hernán M Reeves B Savović J Berkman N Viswanathan M ROBINS-I: a tool for assessing risk of bias in non-randomised studies of interventions. *BMJ.* (2016) 355:i4919. 10.1136/bmj.i4919 27733354 PMC5062054

[16] Sterne JE Moher D. Addressing reporting biases. In: Higgins J Green S editors. *Cochrane Handbook for Systematic Reviews of Intervention. Version 5.1.0.* London: The Cochrane Collaboration (2011).

[17] Momoeda M Akiyama S Yamamoto S Kondo M Fukai T. Burden of menstrual pain measured by heatmap visualization of daily patient-reported data in Japanese patients treated with ethinylestradiol/drospirenone: a randomized controlled study. *Int J Womens Health.* (2020) 12:175–85. 10.2147/IJWH.S242864 32210639 PMC7071861

[18] Strowitzki T Kirsch B Elliesen J. Efficacy of ethinylestradiol 20 μg/drospirenone 3 mg in a flexible extended regimen in women with moderate-to-severe primary dysmenorrhoea: an open-label, multicentre, randomised, controlled study. *J Fam Plann Reprod Health Care.* (2012) 38:94–101. 10.1136/jfprhc-2011-100225 22454006 PMC3353877

[19] EU Clinical Trials Register. *A Multi-Center, Open-Label, Randomized, Controlled, Parallel-Group Study to Assess Efficacy and Safety of an Extended Flexible Regimen of the Combined Oral Contraceptive SH T00186d (0.02 Mg Ethinylestradiol as Beta-Cyclodextrin Clathrate and 3 Mg Drospirenone) Compared to the Conventional Regimen of SH T00186D in the Treatment of Primary Dysmenorrhea SH T00186 in the Treatment of Primary Dysmenorrhea SH T00186 in the Treatment of Primary Dysmenorrhea*. (2006). Available online at: https://www.clinicaltrialsregister.eu/ctr-search/trial/2006-004899-13/DE (accessed October 29, 2014).

[20] Takeda T Kondo A Koga S Hayakawa J Hayakawa K Hiramatsu K Effectiveness of ethinylestradiol/drospirenone for premenstrual symptoms in Japanese patients with dysmenorrhea: open-label pilot study. *J Obstet Gynaecol Res.* (2015) 41:1584–90. 10.1111/jog.12774 26310836

[21] Tanaka Y Mori T Ito F Koshiba A Kusuki I Kitawaki J. Effects of low-dose combined drospirenone-ethinylestradiol on perimenstrual symptoms experienced by women with endometriosis. *Int J Gynecol Obstet.* (2016) 135:135–9. 10.1016/j.ijgo.2016.05.004 27477035

[22] Taniguchi F Enatsu A Ota I Toda T Arata K Harada T. Effects of low dose oral contraceptive pill containing drospirenone/ethinylestradiol in patients with endometrioma. *Eur J Obstet Gynecol Reprod Biol.* (2015) 191:116–20. 10.1016/j.ejogrb.2015.06.006 26115056

[23] Hauksson A Ekström P Juchnicka E Laudański T Akerlund M. The influence of a combined oral contraceptive on uterine activity and reactivity to agonists in primary dysmenorrhea. *Acta Obstet Gynecol Scand.* (1989) 68:31–4. 10.3109/00016348909087685 2801028

[24] Li X Qian F He Y Zhang X Zhang Y Hou C [Clinical observation of combined oral contraceptives drospirenone and ethinylestradiol tablets (II) in the treatment of dysmenorrhea in Chinese women]. *Zhonghua Fu Chan Ke Za Zhi.* (2021) 56:684–90. 34823317 10.3760/cma.j.cn112141-20210719-00385

[25] Sun X Qian F He Y Gu X Di W. Safety and efficacy of combined oral contraceptive ethinyl estradiol/drospirenone (YAZ) in Chinese women: a single-arm, open-label, multicenter, post-authorization study. *Adv Ther.* (2020) 37:906–17. 10.1007/s12325-019-01210-2 31950432

[26] Yoshino O Suzukamo Y Yoshihara K Takahashi N. Quality of life in Japanese Patients with dysmenorrhea or endometriosis-associated pelvic pain treated with extended regimen ethinylestradiol/drospirenone in a real-world setting: a prospective observational study. *Adv Ther.* (2022) 39:5087–104. 10.1007/s12325-022-02301-3 36053449 PMC9525394

[27] Bachmann G Sulak P Sampson-Landers C Benda N Marr J. Efficacy and safety of a low-dose 24-day combined oral contraceptive containing 20 micrograms ethinylestradiol and 3 mg drospirenone. *Contraception.* (2004) 70:191–8. 10.1016/j.contraception.2004.05.013 15325887

