# Supplementary material for: Effect and safety of drospirenone and ethinylestradiol tablets (II) for dysmenorrhea: A systematic review and meta-analysis
Source: Front Med (Lausanne). 2022 Dec 15;9:938606. doi: 10.3389/fmed.2022.938606 (PMC9799974; doi:10.3389/fmed.2022.938606)

Additional files

Supplementary Methods. Search strategies

Supplementary Table 1. Characteristics of included studies

Supplementary Table 2. Total efficiency rate reported in comparison of Drospirenone and Ethinylestradiol Tablets (II) versus control

Supplementary Table 3. Adverse events reported in comparison of Drospirenone and Ethinylestradiol Tablets (II) versus control

Supplementary Table4. Adverse events reported in comparison of Drospirenone and Ethinylestradiol Tablets (II) flexible extended regimen versus conventional 24/4-day regimen

Supplementary Table 5. Adverse events reported in single-arm studies

Supplementary Figure 1. Drospirenone and Ethinylestradiol Tablets (II) versus control: meta-analysis of VAS score for dysmenorrhea

Supplementary Figure 2. Drospirenone and Ethinylestradiol Tablets (II) flexible extended regimen versus conventional 24/4-day regimen: meta-analysis of number of days of dysmenorrhea

Supplementary Figure 3. Drospirenone and Ethinylestradiol Tablets (II) flexible extended regimen versus conventional 24/4-day regimen: meta-analysis of the risk of headache

Supplementary Methods. Search strategies

A. Pubmed

1. ethinylestradiol/drospirenone

2. ethinylestradiol 20 μg/drospirenone 3 mg

3. drospirenone plus ethinylestradiol

4. ethinylestradiol 20 μg plus drospirenone 3 mg

5. ee 20 μg/drospirenone 3 mg

6. 24/4 regimen

7. drospirenone/ethinyl estradiol

8. ee 20 μg/drsp 3mg

9. ee/drospirenone

10. yaz

11. 3 mg drospirenone/20 microg ethinylestradiol

12. 3-milligram drospirenone/20-microgram ethinylestradiol

13. 20 mug ethinylestradiol and 3 mg drospirenone

14. 1 or 2 or 3 or 4 or 5 or 6 or 7 or 8 or 9 or 10 or 11 or 12 or 13

15. dysmenorrhea

16. menstrual pain

17. pelvic pain

18. painful menses

19. dyspareunia

20. 15 or 16 or 17 or 18 or 19

21. 14 and 20

B. EMBASE and CENTRAL（Ovid）

1. ethinylestradiol drospirenone.mp. or ethinylestradiol drospirenone/

2. drospirenone plus ethinylestradiol.mp. or drospirenone plus ethinylestradiol/

3. yaz.mp. or yaz/

4. 24 4 regimen.mp. or 24 4 regimen/

5. ethinylestradiol 20 mug drospirenone 3 mg.mp. or ethinylestradiol 20 mug drospirenone 3 mg/

6. "20 mug ethinylestradiol and 3 mg drospirenone".mp. or "20 mug ethinylestradiol and 3 mg drospirenone"/

7. drospirenone ethinyl estradiol.mp. or drospirenone ethinyl estradiol/

8. ee drospirenone.mp. or ee drospirenone/

9. 1 or 2 or 3 or 4 or 5 or 6 or 7 or 8

10. dysmenorrhea.mp. or dysmenorrhea/

11. menstrual pain.mp. or menstrual pain/

12. pelvic pain.mp. or pelvic pain/

13. painful menses.mp. or painful menses/

14. dyspareunia.mp. or dyspareunia/

15. 10 or 11 or 12 or 13 or 14

16. 9 and 15

C. Cochrane

1. ethinylestradiol drospirenone

2. drospirenone plus ethinylestradiol

3. yaz

4. "24 4 regimen"

5. ethinylestradiol 20 mug drospirenone 3 mg

6. "20 mug ethinylestradiol and 3 mg drospirenone"

7. drospirenone ethinyl estradiol

8. ee drospirenone

9. ethinylestradiol 20 µg drospirenone 3 mg

10. ethinylestradiol 20 µg plus drospirenone 3 mg

11. ee 20 µg drospirenone 3 mg

12. ee 20 µg drsp 3mg

13. 3 mg drospirenone 20 microg ethinylestradiol

14. 3 milligram drospirenone 20 microgram ethinylestradiol

15. 20 mug ethinylestradiol and 3 mg drospirenone

16. 1 or 2 or 3 or 4 or 5 or 6 or 7 or 8 or 9 or 10 or 11 or 12 or 13 or 14 or 15

17. MeSH descriptor: [Dysmenorrhea] explode all trees

18. MeSH descriptor: [Pelvic Pain] explode all trees

19. MeSH descriptor: [Dyspareunia] explode all trees

20. dysmenorrhea

21. menstrual pain

22. pelvic pain

23. painful menses

24. dyspareunia

25. 17 or 18 or 19 or 20 or 21 or 22 or 23 or 24

26. 16 and 25

D. CNKI

SU=(屈螺酮炔雌醇+优思悦+YAZ) AND SU=(痛经+经行腹痛+盆腔痛+盆腔疼痛+性交痛)

E. Wanfang Data

主题:("屈螺酮炔雌醇" or "优思悦" or "YAZ") and 主题:("痛经" or "盆腔疼痛" or "性交痛")

F. CBM

("屈螺酮炔雌醇"[常用字段:智能] OR "优思悦"[常用字段:智能] OR "YAZ"[常用字段:智能]) AND ("痛经"[常用字段:智能] OR "盆腔痛"[常用字段:智能] OR "盆腔疼痛"[常用字段:智能] OR "性交痛"[常用字段:智能])

G. VIP

(((任意字段=屈螺酮炔雌醇 OR 任意字段=优思悦) OR 任意字段=Yaz) AND ((((任意字段=痛经 OR 任意字段=行经腹痛) OR 任意字段=盆腔痛) OR 任意字段=盆腔疼痛) OR 任意字段=性交痛))

H. web of science

1. TS=(ethinylestradiol/drospirenone OR ethinylestradiol 20 μg/drospirenone 3 mg OR drospirenone plus ethinylestradiol OR ethinylestradiol 20 μg plus drospirenone 3 mg OR ee 20 μg/drospirenone 3 mg OR 24/4 regimen OR drospirenone/ethinyl estradiol OR ee 20 μg/drsp 3mg OR ee/drospirenone OR yaz OR 3 mg drospirenone/20 microg ethinylestradiol OR 3-milligram drospirenone/20-microgram ethinylestradiol OR 20 mug ethinylestradiol and 3 mg drospirenone)

2. TS=(dysmenorrhea OR menstrual pain OR pelvic pain OR painful menses OR dyspareunia)

3. 1 AND 2

I. SCOPUS

1. TITLE-ABS-KEY("ethinylestradiol/drospirenone" OR "ethinylestradiol 20 µg/drospirenone 3 mg" OR "drospirenone plus ethinylestradiol" OR "ethinylestradiol 20 µg plus drospirenone 3 mg" OR "ee 20 µg/drospirenone 3 mg" OR "24/4 regimen" OR "drospirenone/ethinyl estradiol" OR "ee 20 µg/drsp 3mg" OR "ee/drospirenone" OR yaz OR "3 mg drospirenone/20 microg ethinylestradiol" OR "3-milligram drospirenone/20-microgram ethinylestradiol" OR "20 mug ethinylestradiol and 3 mg drospirenone")

2. TITLE-ABS-KEY(dysmenorrhea OR menstrual pain OR pelvic pain OR painful menses OR dyspareunia)

3. 1 and 2

Supplementary Table 1. Characteristics of included studies

Included studies	Study design	Country	Number of centers	Study subjects	Funding support	Follow-up period	Number of patients at randomization (n)	Age (years)	BMI (kg/m^2)	Menstrual cycle length (days)	Duration of menstrual bleeding (days)
Al-Jefout 2016	Non-randomized controlled trial	Jordan	2	PD patients	No funding	6 months	43	20.2	21.8	27.7	5.0
Harada 2017	RCT	Japan	32	Endometriosis patients	Bayer Yakuhin, Ltd.	24 weeks	312	35.2	21.2	NR	NR
Liu 2019	RCT	China	1	PD patients	Fuzhou Science and Technology Bureau Funding Program<br>Fujian Provincial Natural Guidance Funding Program	6 cycles	231	24.7	21.9	28.9	NR

Momoeda 2020	Single-arm	Japan	33	Dysmenorrhea patients (PD+SD)	Bayer Yakuhin, Ltd.	6 to 8 cycles	531	30.1	NR	NR	NR
NCT00461305 2007	RCT	Japan	26	Dysmenorrhea patients (PD+SD)	Bayer Yakuhin, Ltd.	24 weeks	420	29.3	20.7	29.5	NR
NCT00511797 2007	RCT	Japan	12	Dysmenorrhea patients (PD+SD)	Bayer	16 weeks	124	30.9	20.9	28.5	NR
NCT01892904 2017	RCT	Japan	8	Dysmenorrhea patients (PD+SD)	Bayer Yakuhin, Ltd.	24 weeks	216	29.7	20.7	NR	NR
Strowitzki 2012	RCT	Germany/UK	29	Moderate to severe PD patients	Bayer Health Care Pharmaceuticals, Berlin, Germany	20 weeks	231	25.5	22.4	NR	NR
Takeda 2015	Single-arm	Japan	4	Dysmenorrhea patients with PMS/PMDD	Bayer Yakuhin, Ltd., Osaka, Japan.	6 cycles	48	31.9	21.1	NR	NR

				symptoms (unknown)							
Tanaka 2016	Single-arm	Japan	1	Endometriosis patients	Bayer Yakuhin, Ltd., Osaka, Japan.	6 months	46	34.4	20.8	27.9	NR
Taniguchi 2015	Single-arm	Japan	7	Endometriosis patients	Bayer Yakuhin, Ltd., Osaka, Japan.	6 cycles	49	33.3	20.3	28.8	NR

Note: PD: primary dysmenorrhea, SD: secondary dysmenorrhea, PMS: premenstrual syndrome, PMDD: premenstrual dysphoric disorder, NR: not reported. Age, BMI, menstrual cycle, and duration of menstrual bleeding are expressed as means.

Supplementary Table 2. Total efficiency rate reported in comparison of Drospirenone and Ethinylestradiol Tablets (II) versus control (Liu 2019, RCT)

Intervention	Control	Total efficiency rate<br>Number of events/ total population (%)		RR (95%CI)	*P*-value
		Intervention group	Control group		
Drospirenone and Ethinylestradiol Tablets (II) conventional 24/4-day regimen	Placebo	49/53(92.5)	5/30(16.7)	5.55(2.48–12.39)	<0.0001
Drospirenone and Ethinylestradiol Tablets (II) conventional 24/4-day regimen	Active control drugs	49/53(92.5)	138/148(93.2)	0.99(0.91–1.08)	0.85

The criteria of clinical response were: (1) marked improvement: significant improvement in symptoms and dysmenorrhea was absent; (2) slight improvement: significant alleviation of symptoms, significant reduction in pain, and be free from analgesics; and (3) failure; no improvement or worsening of symptoms. Total efficiency rate = marked improvement + slight improvement; RR: risk ratio.

Supplementary Table 3. Adverse events reported in comparison of Drospirenone and Ethinylestradiol Tablets (II) versus control

Study ID	Follow-up	Type	Intervention	Control	Specific description	Number of events/ total		RR (95%CI)	*P*-value
						Intervention group	Control group		
Al-Jefout 2016 (non-randomized controlled trial)	6 months	SE	Drospirenone and Ethinylestradiol Tablets (II) conventional 24/4-day regimen	NET-A 5 mg/d continuous regimen	Mood changes	3/18	6/ 20	2.22(0.46–10.72)	0.35
					Spotting	6/18	2/ 20	3.33(0.77–14.47)	0.11
					Headache	4/18	2/ 20	0.74(0.14–3.94)	0.32
					Breast tenderness	7/18	2/ 20	1.33(0.52–3.42)	0.06
Harada 2017 (RCT)	24 weeks	AE	Drospirenone and Ethinylestradiol Tablets (II) flexible extended regimen	Placebo	Headache	22/130	20/ 128	1.08(0.62–1.89)	0.78
					Nausea	15/130	6/ 128	2.46(0.99–6.14)	0.05
					Breast pain	3/130	3/ 128	0.98(0.20–4.79)	0.98
					Vomiting	3/130	3/ 128	0.98(0.20–4.79)	0.98
Liu 2019 (RCT)	6 cycles	AE	Drospirenone and Ethinylestradiol Tablets (II) conventional	Placebo	Overall adverse events	13/53	2/ 30	3.68(0.89–15.22)	0.07
					Nausea/vomiting	8/53	2/ 30	2.26(0.51–9.98)	0.28
					Breast tenderness	3/53	0/ 30	4.02(0.21–75.26)	0.35
					Spotting	2/53	0/ 30	2.87(0.14–57.89)	0.49

			24/4-day regimen	Active control drugs	Overall adverse events	13/53	66/ 148	0.55(0.33–0.91)	0.02
					Nausea/vomiting	8/53	43/ 148	0.54(0.27–1.09)	0.08
					Breast pain	3/53	43/ 148	0.64(0.19–2.17)	0.48
					Spotting	2/53	43/ 148	0.47(0.11–2.01)	0.31
NCT00461305 2007 (RCT)	6 cycles	AE	Drospirenone and Ethinylestradiol Tablets (II) conventional 24/4-day regimen	Drospirenone/ethinylestradiol tablets 3 mg/30 µg conventional 24/4-day regimen	Nausea	115/349	25/ 65	0.86(0.61–1.21)	0.38
					Breast discomfort	13/349	5/ 65	0.48(0.18–1.31)	0.15
					Vomiting	18/349	4/ 65	0.84(0.29–2.40)	0.74
					Headache	185/349	39/ 65	0.88(0.71–1.10)	0.27
NCT00511797 2007 (RCT)	4 cycles	AE	Drospirenone and Ethinylestradiol Tablets (II) conventional 24/4-day regimen	Placebo	Nausea	22/62	17/ 62	1.29(0.76–2.19)	0.34
					Headache	30/62	23/ 62	1.30(0.86–1.97)	0.21

Note: RR: risk ratio; SE: side effect, AE: adverse event.

Supplementary Table4. Adverse events reported in comparison of Drospirenone and Ethinylestradiol Tablets (II) flexible extended regimen versus conventional 24/4-day regimen

**Study ID**	**Follow-up**	**Type**	**Specific description**	**Number of events/ total**		**RR (95%CI)**	***P*-value**
				**Flexible$_{MIB}$**	**Conventional**		
NCT01892904 2017 (RCT)	140 days	TEAE	Headache	23/105	22/ 107	1.07(0.63–1.79)	0.81
			Nausea	11/105	8/ 107	1.40(0.59–3.34)	0.45
Strowitzki 2012 (RCT)	140 days	AE	Headache	6/115	20/ 108	0.28(0.12–0.67)	0.004
			Breast pain	3/115	6/ 108	0.47(0.12–1.83)	0.28
			Vomiting	2/115	3/ 108	0.63(0.11–3.67)	0.60

Note: RR: risk ratio; TEAE: treatment-emergent adverse events, AE: adverse event.

Supplementary Table 5. Adverse events reported in single-arm studies

Study ID	Follow-up	Type	Specific description	Incidence
				Number of events/ total (%)
Takeda 2015	6 cycles	AE	Spotting	1/39(2.6)
Tanaka 2016	6 cycles	AE	Nausea	2/46(4.3)
			Headache	2/46(4.3)

Note: AE: adverse event.

Supplementary Figure 1. Drospirenone and Ethinylestradiol Tablets (II) versus control: meta-analysis of VAS score for dysmenorrhea (RCTs)

	DRSP 3 mg/EE 20 µg			Control				Std. Mean Difference
Study or Subgroup	Mean	SD	Total	Mean	SD	Total	Weight	IV, Fixed, 95% CI
**1.2.1 DRSP 3 mg/EE 20 µg vs. Placebo**								
Liu 2019	-3.79	2.3	26	-0.57	2.3	30	33.6%	-1.38 [-1.97, -0.79]
NCT00511797 2007	-31.8	23.55	51	-10.3	24.53	46	66.4%	-0.89 [-1.31, -0.47]
**Subtotal (95% CI)**			**77**			**76**	**100.0%**	**-1.05 [-1.39, -0.71]**
Heterogeneity: Chi² = 1.79, df = 1 (P = 0.18); I² = 44%								
Test for overall effect: Z = 6.06 (P < 0.00001)								
**1.2.2 DRSP 3 mg/EE 20 µg vs. Active control**								
Liu 2019	-3.79	2.3	27	-3.77	2	148	100.0%	-0.01 [-0.42, 0.40]
**Subtotal (95% CI)**			**27**			**148**	**100.0%**	**-0.01 [-0.42, 0.40]**
Heterogeneity: Not applicable								
Test for overall effect: Z = 0.05 (P = 0.96)								

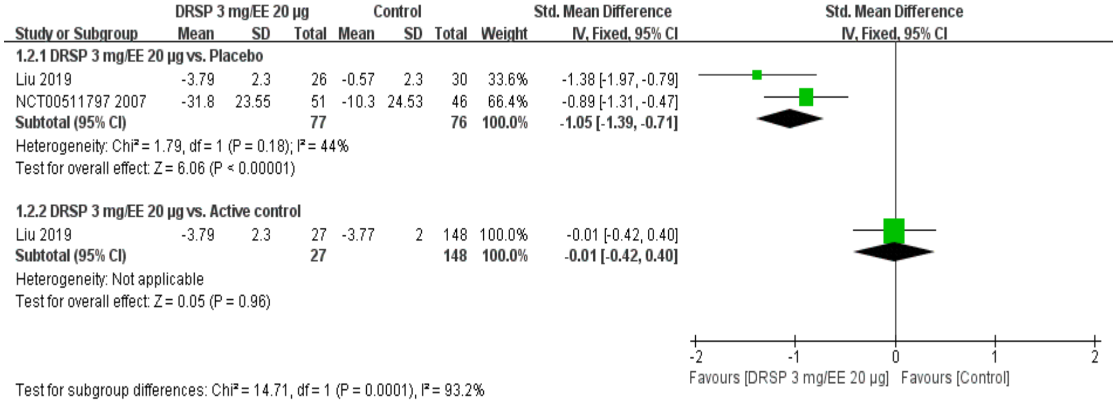

Test for subgroup differences: Chi² = 14.71, df = 1 (P = 0.0001), I² = 93.2%

Supplementary Figure 2. Drospirenone and Ethinylestradiol Tablets (II) flexible extended regimen versus conventional 24/4-day regimen: meta-analysis of number of days of dysmenorrhea (RCTs)

	Flexible extended regimen			Conventional 28d regimen				Mean Difference
Study or Subgroup	Mean	SD	Total	Mean	SD	Total	Weight	IV, Random, 95% CI
NCT01892904 2017	11.9	9.4	105	15.3	11.8	107	35.5%	-3.40 [-6.27, -0.53]
Strowitzki 2012	10.6	6.7	112	14.9	8.9	102	64.5%	-4.30 [-6.43, -2.17]
**Total (95% CI)**			**217**			**209**	**100.0%**	**-3.98 [-5.69, -2.27]**

Heterogeneity: Tau² = 0.00; Chi² = 0.24, df = 1 (P = 0.62); I² = 0%
Test for overall effect: Z = 4.57 (P < 0.00001)

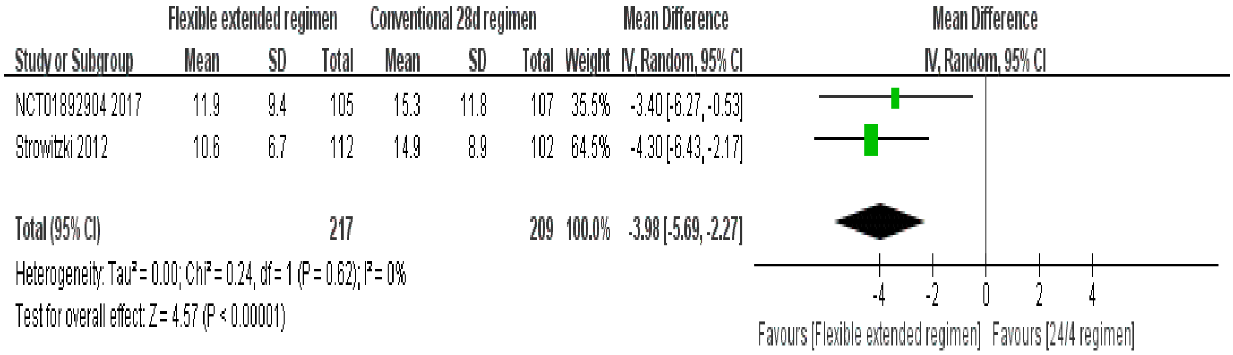

Supplementary Figure 3. Drospirenone and Ethinylestradiol Tablets (II) flexible extended regimen versus conventional 24/4-day regimen: meta-analysis of the risk of headache (RCTs)

	Flexible extended regimen		Conventional 28d regimen			Risk Ratio
Study or Subgroup	Events	Total	Events	Total	Weight	M-H, Random, 95% CI
NCT01892904 2017	23	105	22	107	53.5%	1.07 [0.63, 1.79]
Strowitzki 2012	6	115	20	108	46.5%	0.28 [0.12, 0.67]
**Total (95% CI)**		**220**		**215**	**100.0%**	**0.57 [0.15, 2.14]**
Total events	29		42			

Heterogeneity: Tau² = 0.77; Chi² = 6.76, df = 1 (P = 0.009); I² = 85%
Test for overall effect: Z = 0.83 (P = 0.41)

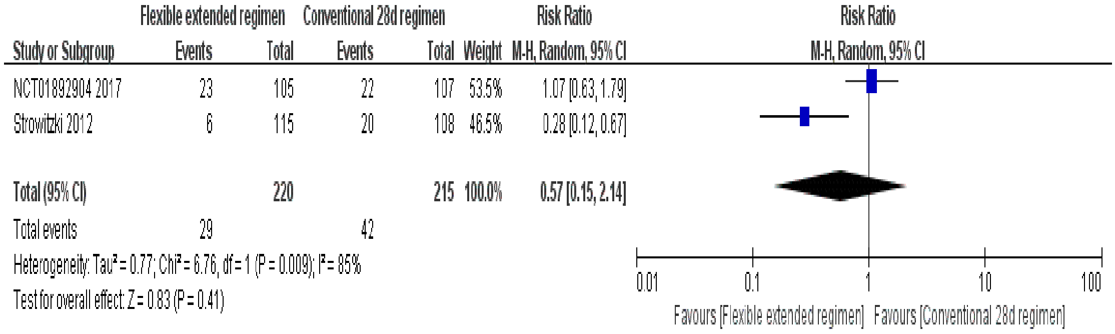

Supplement: Supplementary file 1 [file Data_Sheet_1.pdf]
